# Chemical Characterization and Anti-Oomycete Activity of *Laureliopsis philippianna* Essential Oils against *Saprolegnia parasitica* and *S. australis*

**DOI:** 10.3390/molecules20058033

**Published:** 2015-05-05

**Authors:** Alejandro Madrid, Patricio Godoy, Sebastián González, Luis Zaror, Alejandra Moller, Enrique Werner, Mauricio Cuellar, Joan Villena, Iván Montenegro

**Affiliations:** 1 Departamento de Química, Facultad de Ciencias Naturales y Exactas, Universidad de Playa Ancha, Avda. Leopoldo Carvallo 270, Playa Ancha, Valparaíso 2340000, Chile; 2 Instituto de Microbiología Clínica, Facultad de Medicina, Universidad Austral de Chile, Los Laureles s/n, Isla Teja, Valdivia 5090000, Chile; E-Mails: patricio.godoy@uach.cl (P.G.); floydkimura@gmail.com (S.G.); 3 Escuela de Tecnología Médica, Facultad de Medicina, Universidad Mayor, Av. Alemania 0281, Temuco 4780000, Chile; E-Mail: lzaror@yahoo.es; 4 Centro de Investigaciones Biomédicas (CIB), Escuela de Medicina, Universidad de Valparaíso, Av. Hontaneda N° 2664, Valparaíso 2340000, Chile; E-Mails: alemoller_diaz@hotmail.com (A.M.); juan.villena@uv.cl (J.V.); 5 Departamento de Ciencias Básicas, Campus Fernando May, Universidad del Bío-Bío, Avda. Andrés Bello s/n casilla 447, Chillán 3780000, Chile; E-Mail: ewerner@ubiobio.cl; 6 Facultad de Farmacia, Escuela de Química y Farmacia, Universidad de Valparaíso, Av. Gran Bretaña N° 1093, Valparaíso 2340000, Chile; E-Mail: mauricio.cuellar@uv.cl; 7 Escuela de Obstetricia y Puericultura, Facultad de Medicina, Universidad de Valparaíso, Blanco N° 1911, Valparaíso 2340000, Chile

**Keywords:** *Laureliopsis philippianna*, essential oil, *Saprolegnia*, anti-oomycete

## Abstract

*Laureliopsis philippiana* (Looser) R. Schodde (Monimiaceae) is a native tree widespread in the forest areas in the south of Chile and Argentina, known for its medicinal properties and excellent wood. The aim of this study was to evaluate the chemical composition of *L. philippiana* leaf and bark essential oils (EOs) using gas chromatography-mass spectrometry (GC-MS), and to quantify its anti-oomycete activity, specifically against *Saprolegnia* *parasitica* and *S. australis*. Only six components were identified in leaf EO, 96.92% of which are phenylpropanoids and 3.08% are terpenes. As for bark EO, 29 components were identified, representing 67.61% for phenylpropanoids and 32.39% for terpenes. Leaf EO was characterized mainly by safrole (96.92%) and β-phellandrene (1.80%). Bark EO was characterized mainly by isosafrole (30.07%), safrole (24.41%), eucalyptol (13.89%), methyleugenol (7.12%), and eugenol (6.01%). Bark EO has the most promising anti-*Saprolegnia* activity, with a minimum inhibition concentration (MIC) value of 30.0 µg/mL against mycelia growth and a minimum fungicidal concentration (MFC) value of 50.0 μg/mL against spores; for leaf EO, the MIC and MFC values are 100 and 125 µg/mL, respectively. These findings demonstrate that bark EO has potential to be developed as a remedy for the control of *Saprolegnia* spp. in aquaculture.

## 1. Introduction

*Saprolegnia* species, commonly known as water molds (Saprolegniaceae, Saprolegniales, oomycetes), are widespread in fresh water and represent the most significant infections affecting wild and cultured shellfish and fish [1,2]. In the shellfish industries, such as crayfish and fish farms (especially in salmonid species [3,4] and catfish species [5]), the disease affects mainly broodfish and incubating eggs, causing major economic losses worldwide [6,7]. The most aggressive and frequent species of *Saprolegnia* in both Chilean and global aquaculture industries are *S. parasitica* and *S. australis*. *S. parasitica* is endemic to all fresh water habitats around the world and is partly responsible for the decline of natural populations of salmonids and other fresh water fish [8]. Besides being a problem for the fish farming industry, *S. parasitica* has also been implicated in wild salmon population decline around the world [9]. *S. parasitica* infections have also been reported in crayfish, both in aquaculture settings and in wild crayfish [10]. Of note, *S. australis* is closely related to *S. parasitica*. Diseases involving *S. australis* and related organisms have been described in crayfish from Lake Constance in Europe and Big Muskellunge Lake in the USA [10]. Traditionally, *Saprolegnia* spp. over the years infections were effectively controlled with various synthetic fungicides (such as malachite green, formalin, or hydrogen peroxide) and biocides (bronopol) in aquaculture industries; however, the use of these types of compounds has led to a number of problems, including the development of fungicide resistance and potentially harmful effects to human health [11,12]. In addition, the increasing public concerns over fungicide safety and possible damages to the environment have resulted in increased attention given to natural products for use in control and prevention of the disease [13,14]. In this context, a well-known way to reduce the proliferation of microorganisms is through the use of essential oils (EOs) or plant extracts [15], due to a lesser chance of their being toxic. In this context, many plant products have been evaluated for their toxic properties against different aquatic fungi [16,17,18], especially in the form of EOs [19]. EOs have proven to be inhibitory against *Saprolegnia* spp., depending upon their concentration, method of testing, and the active constituents present [20,21,22].

*Laureliopsis philippiana* (Looser) R. Schodde (synonym *Laurelia philippiana*) is a native tree, widespread in the forest areas of the south of Chile and Argentina [23]. It is known by the vernacular names “Tepa” or “Huanhuán”, and extracts of its leaves, flowers, and bark have traditionally been used by the Chilean Mapuche people as expectorant, anti-inflammatory, and for the treatment of colds and headaches [24]. As a result, chemical characterization has been focused on compounds with medicinal applications. Phytochemical studies of leaves and stem bark of *L. philippiana* have revealed high contents of aporphinoid and bisbenzylsoquinoline alkaloids [25,26,27]. Another study investigated the insecticide and repellent properties against *Sitophilus zeamais* Motschulsky (Coleoptera) of powder derived from leaves of *L. philippiana* [28]. In addition, previous studies have determined fungicidal and insecticidal activity of EO from leaves of *L. philippiana* [29,30]. There is no information concerning the efficacy of EOs of the *L. philippiana* against aquatic fungi, in particular *Saprolegnia* spp., in Chile. The aim of this study was to characterize the chemical composition of *L. philippiana* leaf and bark EOs using GC-MS and evaluate them for their anti-oomycete activities against *S. parasitica* and *S. australis*.

## 2. Results and Discussion

### 2.1. Chemical Composition of EOs

The extraction yield of *L. philippiana* EOs was 2.47% and 5.26% (v/dry weight) and the density was 1.11 ± 0.02 and 0.982 ± 0.02 g/mL, for leaf and bark EOs, respectively. The results of the gas chromatographic analysis of *L. philippiana* EOs are summarized in Table 1.

**Table 1:** Main components of *L. philippianna* leaf and bark essential oils (EOs).

Main Components	RI	Leaf EO	Bark EO
		% Area	% Area
α-Pinene	923	0.88	0.96
Camphene	937	- ^a^	0.39
Sabinene	962	-	0.44
β-Pinene	966	-	1.73
α-Phellandrene	996	1.80	4.69
α-Terpinene	1008	0.17	0.50
Limonene	1019	-	0.49
1,8-Cineol	1022	-	0.57
Eucaliptol	1035	-	13.89
*trans*-β-Ocimene	1036	0.14	0.51
γ-Terpinene	1050	-	0.34
Camphor	1134	-	0.21
Borneol	1158	-	0.49
Isosafrole	1296	-	30.07
Safrole	1307	96.92	24.41
Eugenol	1327	-	6.01
α-Cubenene	1346	-	0.18
Methyleugenol	1374	-	7.12
α-Copaene	1391	-	0.26
Decyl acetate	1412	-	0.24
Gurjunene	1423	-	0.51
2-Norpinene	1436	-	0.33
Alloaromadendrene	1455	-	2.37
*trans*-γ-Cadinene	1496	-	0.35
α-Cuparene	1515	-	1.14
Caryophyllene oxide	1575	-	0.54
Isoelemicin	1596	-	0.37
β-Eudesmol	1681	0.09	0.04
Squalene	2847	-	0.85

^a^ - indicates “not found”.

Only six components were identified in the leaf EO: 96.92% were phenylpropanoids and 3.08%, terpenes. On the other hand, 29 components were identified in the bark EO, corresponding to 67.61% phenylpropanoids and 32.39% terpenes. Leaf EO was mainly characterized by safrole (96.92%) and β-phellandrene (1.80%). Bark EO was mainly characterized by isosafrole (30.07%), safrole (24.41%), eucalyptol (13.89%), methyleugenol (7.12%), and eugenol (6.01%). The chemical compositions of leaf and bark EOs differ according to the amount of certain major components, such as safrole.

Some studies of leaf EOs from *L. philippiana* showed different chemical compositions according to the region. Results of this work showed that the chemical composition of these EOs was different with those obtained from plants collected in the Bio-Bío and La Araucanía regions [30,31]. The most important differences are the lower content of 3-carene and eucalyptol, and the higher content of safrole. This variability can reflect the influence of extrinsic conditions based on geographic origin (different climatic and soil-growth conditions), the age of the plant, method of drying and method of extraction of the oil [32,33]. In addition, this is the first report made on the compositions of EOs derived from cuts of *L. philippiana* in which phenylpropanoids was the predominant components. According to some reports [34,35] differences in chemical composition might suggest the presence of different *L. philippiana* chemotypes.

### 2.2. Anti-Oomycete Susceptibility

The major aim of this study was to investigate the substitution of bronopol with *L. philippiana* natural EOs, or purified compounds, to treat infections of *Saprolegnia* spp. and, if possible, to test the top performing candidates on *S. parasitica* and *S. australis*. EOs used in this study presented activity against the 32 isolates tested. The highest inhibition halo value observed for the leaf EO was against isolate N° 19, with 22.3 mm of diameter, while the minimum value was of 10.7 mm for isolate N° 32. For the bark EO the isolate that presented the most sensitive was N° 19, with an inhibition halo of 18.2 mm. The isolates (N° 1, N° 2 and N° 3) were the most sensitive to bronopol, together with N° 4, and N° 21. When comparing the effectiveness of the oils against the strains, it was observed that both EOs were very efficient for pathogenic isolates (N° 1–30 and N° 32), while the bark EO revealed major efficiency for only 2 strains (N° 31 and N° 32). Both EOs showed bronopol-equivalent effectiveness against strains N°s 8, 9, 12, 14, and 17. As an aside, previous studies have reported that EOs from *Laurelia sempervirens* (Ruiz and Pavón) Tul. (Monimiaceae) and *Drymis winteri* J.R. Forster and G. Forster (Winteraceae) present similar anti-oomycidal activity against *S. parasitica* and *S. australis*. 

The gas chromatographic analysis data of the *L. philippiana* bark and leaves EOs shown in Table 1 indicate a high concentration of phenylpropanoids. The presence of the phenolic content most likely explains the action of both EOs, since there are reports that relate these structures with antimicrobial capacity; an example are bark EOs from *L. sempervirens,* which show a strong effect against multidrug-resistant bacteria [36]. In that EO, safrole was one of the main (65.03%) metabolites in the samples. Furthermore, safrole is the main constituent (74.29%) present in the EO of *Piper auritum* Kunth. As for its effectiveness, Himejima and Kubo reported that safrole possesses moderate activity against *Saccharomyces cerevisiae*, *Candida utilis*, *Pityrosporum ovale*, and *Penicillum* *chrysogenum*, in broth dilution methods (*P. ovale* being the most sensitive fungus thereto) [37]. In a second article, Kubo reported that safrole possessed moderate activity against *Candida albicans* [38]. It has since shown high antimicrobial activity when tested against *Xanthomonas albilineans* and *Acidovorax avenae* subsp. *avenae* [39]. Isosafrole, an isomer of safrole, has two types of isomers and is the precursor to both the illicit drug MDMA and spathulenol (6.60%); this sesquiterpene presents activity against *Escherichia coli*, *Salmonella typhimurium*, and *Staphylococcus aureus* [40]. Eugenol is another phenylpropanoid found in bark EO from *L. philippiana* that shows antifungal activity against phytopathogenic fungi *Alternaria* spp. and *Penicillium chrysogenum* [41].

The minimum inhibition concentration (MIC) [42], can be defined as the minimum concentration of anti-oomycidal agent that is able to inhibit the visible oomycete growth after an incubation period of 48 h. This method has been used and is considered a fundamental instrument for the determination of susceptibility of microorganisms to anti-oomycidal agents. Table 2 presents the values obtained for the MIC for isolates (N° 1–32), which, in the initial screening, presented some susceptibility to EOs. The value of the MIC for bronopol varied between 100 µg/mL and 150 µg/mL. For EOs and safrole (major compound present in leaf EO), the value of MIC ranged between 50 µg/mL and 200 µg/mL. For eugenol (present in bark EO), the MIC values ranged between 100 µg/mL and 200 µg/mL. In previous studies, eugenol exhibited promising activity, with MIC values of 500, 250, and 130 µg/mL against *Saprolegnia* spp. [43]. Indeed, another study [44] showed that eugenol has even better anti-oomycete effects against *S. parasitica*, *S. diclina*, and *S. ferax*.

Of all the samples that were tested (see Table 3), the bark EO had the lowest spore count at the end of the trial and was shown to inhibit growth at concentrations of 50–175 µg/mL for all strains. The leaf EO failed to show an effect in this trial when used at doses less than 100 µg/mL, the leaf EO was shown to inhibit growth at concentrations of 125–200 µg/mL for various strains. This observation may be due to the major percentage of safrole in the leaf composition (see Table 1). The results indicate that anti-oomycete activity was diminished at 72 h post-inoculation. There was a decrease in the absorbance of the solution (data not show), suggesting that the germination of the spores was being inhibited. Since spores are produced within oospores and zoospores hyphae, inhibition of germination leads to a decrease in spore production. These results were compared to bronopol, known for its inhibitory effect on spores. Although the effect of bronopol is better than EOs, this may be due to the solubility of bronopol in water, which is higher than EOs and tested compounds. This experiment was carried out to provide a preliminary assessment of the efficacy of bronopol in highly resistant *S. parasitica*. Bronopol was inactive against strains N° 24–30 and N° 32.

**Table 2:** MIC values ^a^ of leaf and bark EOs against mycelium at 48 h.

N° Strains	EOs		Compounds		
	Leaf	Bark	Safrole	Eugenol	Bronopol
**1**	123 ± 12	30.0 ± 10	130 ± 11	130 ± 4.2	95 ± 5.3
**2**	125 ± 11	33.3 ± 9.0	150 ± 10	132 ± 5.8	98 ± 4.7
**3**	120 ± 15	32.3 ± 8.0	135 ± 8.5	138 ± 4.4	97 ± 6.2
**4**	125 ± 12	31.2 ± 8.5	140 ± 6.5	156 ± 11	100.4 ± 9.4
**5**	130 ± 17	35.4 ± 9.5	125 ± 7.3	125 ± 12	134 ± 9.0
**6**	150 ± 9.0	36.3 ± 9.2	168 ± 6.6	134 ± 9.3	144 ± 13
**7**	140 ± 18	34.3 ± 11	150 ± 12	139 ± 9.1	145 ± 6.6
**8**	>200	60.3 ± 8.5	>200	140 ± 8.2	165 ± 7.4
**9**	100 ± 17	30.0 ± 12	115 ± 12	145 ± 19	165.3 ± 8.6
**10**	150 ± 13	45.3 ± 9.2	155 ± 18	132 ± 6.6	176.4 ± 9.2
**11**	151 ± 16	47.2 ± 16	157 ± 4.8	>200	>200
**12**	125 ± 15	30.0 ± 9.2	143 ± 9.0	150 ± 6.9	>200
**13**	150 ± 16	30.0 ± 8.3	161 ± 12	>200	156.2 ± 7.8
**14**	150.4 ± 14	30.5 ± 8.7	156.6 ± 6.5	>200	167.2 ± 5.8
**15**	152.3 ± 12	30.0 ± 12	160.5 ± 6.7	>200	165.4 ± 7.9
**16**	200 ± 15	30.0 ± 9.0	>200	175 ± 13	200 ± 5.3
**17**	100 ± 14	25.1 ± 4.0	159.2 ± 6.3	134 ± 8.7	156.5 ± 11
**18**	150 ± 8	30.0 ± 4.5	156.7 ± 7.5	>200	200 ± 5.1
**19**	75 ± 17	30.3 ± 4.8	90.3 ± 4.5	100.2 ± 8.4	134.3 ± 3.4
**20**	175 ± 12	35.4 ± 5.0	187.4 ± 10	130.0 ± 10	143.3 ± 4.7
**21**	150 ± 11	30.5 ± 6.0	165.3 ± 17	139 ± 9.0	117.3 ± 7.8
**22**	153 ± 19	31.5 ± 4.5	166.5 ± 19	144 ± 9.9	200 ± 4.3
**23**	132 ± 10	35.6 ± 5.5	145.1 ± 13	145 ± 9.8	123.4 ± 8.7
**24**	>200	65.4 ± 5.3	>200	>200	>200
**25**	>200	130.3 ± 4.8	>200	>200	>200
**26**	>200	140.5 ± 10	>200	>200	>200
**27**	150 ± 11	30.1 ± 4.9	156.2 ± 4.1	>200	>200
**28**	200 ± 20	142.3 ± 7.8	>200	165 ± 10	>200
**29**	185 ± 12	130.3 ± 6.5	>200	187 ± 11	>200
**30**	>200	150.0 ± 4.6	>200	>200	>200
**31**	>200	155.3 ± 8.7	>200	158 ± 6.7	160.3 ± 9.7
**32**	>200	150.6 ± 9.3	>200	>200	>200

^a^ Each value represents the mean ± SD of three experiments, performed in quadruplicate.

The observed results show that EOs of different organs of the same tree can present different modes of action over different time intervals [45,46]. Comparison of spore counts and absorbance values obtained (data not shown), showcases some of the molecules that combat infections caused by *Saprolegnia* and the possibility of synthetic derivatives to enhance the activity.

**Table 3:** MFC values ^a^ of leaf and bark EOs against spores at 72 h.

N° Strains	EOs		Compounds		
	Leaf	Bark	Safrole	Eugenol	Bronopol
**1**	125	50	150	150	125
**2**	150	50	150	150	125
**3**	125	75	150	150	100
**4**	150	75	150	175	125
**5**	150	100	125	150	150
**6**	175	75	175	175	150
**7**	150	50	150	200	175
**8**	>200	100	>200	175	175
**9**	125	50	125	175	175
**10**	175	75	200	150	200
**11**	175	175	200	>200	>200
**12**	125	150	150	200	>200
**13**	150	150	175	>200	175
**14**	175	175	175	>200	200
**15**	175	75	200	>200	>200
**16**	>200	50	>200	200	>200
**17**	125	50	175	150	175
**18**	175	50	175	>200	>200
**19**	100	50	125	150	150
**20**	200	50	200	175	175
**21**	175	175	175	150	125
**22**	175	175	175	150	>200
**23**	150	150	175	150	150
**24**	>200	100	>200	>200	>200
**25**	>200	150	>200	>200	>200
**26**	>200	175	>200	>200	>200
**27**	175	75	175	>200	>200
**28**	>200	150	>200	200	>200
**29**	200	150	>200	200	>200
**30**	>200	175	>200	>200	>200
**31**	>200	175	>200	175	175
**32**	>200	175	>200	>200	>200

^a^ Each value represents the mean ± SD of three experiments, performed in quadruplicate.

In summary, leaf and bark EOs, and safrole in particular, have potential to be developed as a therapy for treatment against *Saprolegnia* infections. However, *in vivo* activity needs to be further investigated, and field studies are required before practical use of the chemical in fish aquaculture.

## 3. Experimental Section

### 3.1. Chemical and Reagents

Safrole (99% pure), eugenol (98% pure), bronopol (99% pure) oxytetracycline (99% pure), flumequine (99% pure), and florfenicol (98% pure) were purchased from Sigma Chemical Co. (St. Louis, MO, USA). Other reagents and solvents were purchased from commercial suppliers and were analytical grade. 

### 3.2. Plant Material

The leaves and bark of *L. philippiana* were collected in Antilhue, Los Ríos Region, Chile, in December 2012. Plant material was identified by Forest Engineer Patricio Novoa (Jardín Botánico Nacional, Viña del Mar, Chile), and a specimen has been deposited at the Herbarium of the Natural Products Laboratory, Department of Chemistry, Universidad de Playa Ancha, Valparaíso, Chile, with voucher number Lp-12112.

### 3.3. Extraction of EOs

The leaves and bark of *L. philippiana* were dried separately at room temperature for five days. Dried plant material were submitted to hydrodistillation with 1 L of distilled water for 4 h in a Clevenger-type apparatus, following which the EO layer was separated, dried over anhydrous sodium sulfate and was stored for some days at −20 °C in amber glass bottles. The yield of fresh EO was determined as the quotient of the weight of oil collected and the dry weight of plant material extracted.

### 3.4. GC/MS Analysis

EOs were diluted with *n*-hexane, and 1 μL of this was sampled for the gas chromatography. Analysis thereof was performed in a HP 6890 chromatograph equipped with a programmable vaporization temperature inlet (PTV). This machine was coupled to an HP 5973 mass-selective detector (Hewlett-Packard, Palo Alto, CA, USA) in scan mode in order to determine the fractional composition of the extracts. The injector temperature was maintained at 280 °C in a pulsed, splitless mode. A GC program temperature ramp was set at 70 °C for 3 min, and then increased at a rate of 10 °C/min. up to 300 °C, to afford the best separation through a capillary HP-5 MS column. The transfer line was maintained at 300 °C. Mass spectra was obtained at 70 eV and compared through direct matching by using a NIST Mass Spectral Search Program and a NIST/EPA/NIH Mass Spectral Library [47]. 

### 3.5. Isolation and Identification of Saprolegnia spp.

For the purposes of this study, *Saprolegnia* spp. were isolated from an infected Atlantic salmon, (*Salmo salar* L.), caught in Puerto Montt, Chile. The isolation procedure was performed according to reported procedures [48]. The culture used was reference type strain ATCC 42062 *S. parasitica* and *S. australis* 38487. Vouchers of the cultures are kept in the fungal culture collections of the Centro de Investigaciones Biomédicas, Escuela de Medicina, Universidad de Valparaíso, Valparaíso, Chile. Small pieces of hyphae were placed on Petri-dishes of yeast dextrose (DY) agar [49], and an antibiotic mixture (oxytetracycline 80%, flumequine 100%, and florfenicol 80%) was added in order to eliminate any accompanying bacterial biota. The cultures were purified by successive transfers (at least five times) using agar pieces (10 × 10 mm) obtained from the periphery of the growing colony. The plates were then incubated at 18 °C and sub-cultured every 3 days. A reference population was established on a DY slope and held at 4 °C.

### 3.6. Molecular Characterization of Saprolegnia spp.

From fresh and pure cultures of *Saprolegnia* spp., small pieces cut from the periphery thereof were incorporated into Eppendorf tubes. DNA, once extracted, was amplified using the following primers: nu-SSU-1766-5' (ITS5) 5'-GGAAGTAAAAGTCGTAACAAGG-3' and nu-LSU-0041-3' (ITS4) 5'-TCCTCCGCTTATTGATATGC-3'. This was to amplify the internal transcribed spacer (ITS1 and ITS2) and 5.8 S rRNA gene, as well as all of the rRNA gene clusters. In relation to genetic variability, to establish the existence of this, RAPD-PCR was performed. In this study, DNA was extracted using the protocol manual [50], although slightly modified, and with the E.Z.N.A. Fungi DNA Miniprep Kit (Omega Biotek, Doraville, GA, USA).

The primers used in the amplification of genomic material [51] are as follows: A04 5'-AATCGGGCTG-3', A07 5'-GAAACGGGTG-3', A09 5'-GGGTAACGCC-3', A10 5'-GTGATCGC AG-3', A12 5'-TCGGCGATAG-3', B01 5'-GTTTCGCTCC-3', B05 5'-TGCGCCCTTC-3', B11 5' GTAGACCCGT-3' and B15 5'-GGAGGGTGTT-3'. RT-Q-PCR, which were performed in a thermocycler (Mx3000P, Stratagene, Santa Clara, CA, USA).

### 3.7. Recovery of Spores Saprolegnia spp. from Suspensions with Known Concentration

To assess the ability of the method to recover spores [52], samples of spore suspensions with predetermined concentrations of 500, 1000, 5000, and 10,000 spores per liter, were used. The spore solutions were made by culturing two *Saprolegnia* strains on Sabouraud (SAB) [4] and glucose-yeast (GY) culturing media with subsequent rinse in autoclaved water from aquarium tanks containing salmonid fish, according to the method of [6].

### 3.8. Microwell Enumeration Method

The method is based on inoculation of water samples in a fluid growth medium, in 96-well microwell plates (MWP) with subsequent enumeration by recording of viable cultures per plate. By using the microwell plates, the sample is dispersed in small volumes for easier segregation of the spores of *Saprolegnia* spp. Inoculation of a sample volume of 100 mL per well in a MWP gives a total of 9.6 mL examined from each sample per plate. The inoculated volume, however, can be adjusted based on the presumed spore load in the sample. Replicate MWP for each sample are used to increase the accuracy of the method [52].

### 3.9. Determination of Minimum Inhibitory Concentration (MIC)

The method used in this study for anti-oomycete activity assay was performed with some modifications of [42]. To summarize the original assay, 80 µL of Griffin’s sporulation medium [53] were added to replicate wells of a flat-bottomed, 96-well polystyrene plate. Each sample [dissolved in ethanol (EtOH)] was serially diluted in 1% aqueous solution of EtOH in water at 10 times the desired test concentrations (200, 150, 125, 100, 75, 50, 25, 12.5, 6.25, and 3.125 µg/mL) to find a preliminary minimum inhibitory concentration (MIC) interval. Ten microliters of each 10-dilution were added to triplicate wells. Then, 10 µL of either zoospore suspension was added to each well. For *Saprolegnia* spp., 100 µL of Gypsum (G-Y) medium was added to all wells 24 h after exposure to the drug. All wells were scored for the presence or absence of water mold growth 48 h after the start of the experiment. All the independent experiments were conducted three times with triplicates at each test concentration. Thus, a 1% aqueous solution of EtOH in water was the negative control, while bronopol was the positive controls.

### 3.10. Spores Germination Inhibition Test

The spore germination inhibition test was carried out by agar dilution method [54]. In brief, the *Saprolegnia* isolate was grown on potato dextrose agar (PDA) plates for 7–14 days, after which time spores were harvested from sporulating colonies and suspended in sterile distilled water (SDW). The concentration of spores in suspension were determined using a hemocytometer and adjusted to 1 × 10^4^ CFU/mL approximately. The agar plates were prepared with the required concentration of the active samples, added to 10 mL of molten PDA (about 65 °C). Ten microliters of the spore suspension were spotted in triplicate on these plates which were then incubated at 25 °C for 72 h. The minimum oomyceticidal concentration (MOC) was defined as the lowest concentration of the chemicals that prevented visible growth or germination of spores.

### 3.11. Mycelial Growth Inhibition Test

*In vitro* anti-oomycete activities of the natural and synthetic samples were assessed on the basis of mycelia growth inhibition rate [54]. Each sample was added to assay flasks containing hot sterilized PDA (about 65 °C) and final concentrations were adjusted to corresponding concentrations based on the initial tests. After mixing with a vortex, aliquots (10 mL) of treated medium were poured into 7 cm diameter Petri dishes. The *Saprolegnia*-colonized rapeseeds were inoculated in the center of the prepared media. The mycelial growth diameter was measured after inoculation at 25 °C for 48 h. The growth inhibition rate was calculated from mean values as:

%IR = 100 (x − y)/(x − z)
(1)

where IR is the growth inhibition rate; x, the mycelial growth in control; y, the mycelia growth in sample; and z, the average diameter of the rapeseeds.

### 3.12. Statistical Analysis

Statistical analysis of recovery rates was performed by comparison within isolates and between culturing media used with a Student’s t-test. Differences were considered significant at *p* < 0.05.

## 4. Conclusions

The results obtained in this work reveal a high susceptibility of *Saprolegnia* spp. strains to *L. philippianna* EOs. In general, EOs showed higher inhibition halo values as compared to tested isolates. For the majority of the isolates tested, bark EO was more efficient than bronopol, leaf EO, and phenylpropanoids. In the event that EOs do replace synthetic compounds, hatchery operating costs are highly likely to be reduced due to the low cost with which these EOs can be obtained, not to mention that they are also safer for employees. Moreover, use of these EOs in place of bronopol or formalin will relieve some of the environmental pressure on the fish farm industry, which in recent years has been encouraged to pollute less in its operations and reduce its impact in the environment.
